# Serological and clinical associations of autoantibodies in Chinese patients with new-onset systemic lupus erythematosus

**DOI:** 10.1038/s41598-023-37100-5

**Published:** 2023-06-21

**Authors:** Muxue Gong, Li Dai, Zhuobei Xie, Dengxiao Hong, Ning Li, Xiaoyun Fan, Changhao Xie

**Affiliations:** 1 Department of Rheumatology and Immunology, The First Affiliated Hospital of Bengbu Medical College, Bengbu, 233004 China; 2 Anhui Provincial Key Laboratory of Immunology in Chronic Diseases, Bengbu Medical College, Bengbu, 233003 China

**Keywords:** Immunology, Rheumatology, Signs and symptoms

## Abstract

To study the clinical significance of autoantibodies in Chinese patients with new-onset systemic lupus erythematosus (SLE), we enrolled 526 new-onset patients who met the 1997 Updated American College of Rheumatology SLE Classification Criteria for a retrospective cohort study. Chi-square test and Wilcoxon rank-sum test were used to detect the relationship of autoantibodies with clinical manifestations and serological results respectively. Our results demonstrated that the positive rate of anti-ribosomal P protein (anti-P) antibody in female patients was higher than that in male patients (41.2% vs. 22%, *P* = 0.008). Patients with anti-SSB (43.95 ± 73.12 vs. 40.92 ± 75.75, *P* = 0.004; 63.93 ± 103.56 vs. 55.06 ± 120.84, *P* = 0.008 respectively) antibodies had higher levels of alanine aminotransferase (ALT) and aspartate transaminase (AST), whereas those with anti-P antibody (28.90 ± 25.70 vs. 50.08 ± 93.00, *P* = 0.014; 38.51 ± 48.19 vs. 69.95 ± 142.67, *P* = 0.047, respectively) had lower levels of them. Anti-dsDNA antibody (*P* = 0.021) was associated with pulmonary arterial hypertension (PAH). The patients with anti-Ro60 (*P* = 0.044), anti-P (*P* = 0.012) and anti-dsDNA (*P* = 0.013) antibodies were less likely to develop Interstitial lung disease. Anti-SmRNP antibody was correlated to lower prevalence of neuropsychiatric symptoms (*P* = 0.037), and patients with anti-centromere antibody (ACA) were more likely to develop serositis (*P* = 0.016).We identified five clusters of SLE-related autoantibodies, confirmed previously reported associations of autoantibodies, and discovered new associations.

## Introduction

Systemic lupus erythematosus (SLE) is an autoimmune disease related to genetic as well as environmental factors like viral infection and drugs, which induce production of specific autoantibodies. The immunocomplexes formed by these autoantibodies with autoantigens are deposited in capillaries, leading to systemic injuries^1,2^. Therefore, SLE has a broad spectrum of clinical manifestations^3^. However, the exact pathological basis of SLE remains unclear.

Some autoantibodies show high diagnostic sensitivity and specificity for SLE. For instance, anti-nuclear antibody (ANA), anti-double stranded DNA (anti-dsDNA) antibody and anti-Sm antibody have been included in the American College of Rheumatology (ACR) as diagnostic markers of SLE^4^. Furthermore, some of these autoantibodies are directly related to clinical manifestation. For e.g., the anti-dsDNA antibodies have been linked with nephritis^5^, and anti-RNP antibodies with Reynold’s phenomenon^6^. Therefore, a greater understanding of the relationship between autoantibodies and clinical manifestations can help predict organ injury and identify the SLE patients with high risk of developing complication for timely intervention. There are much more researches on anti-dsDNA, anti-Sm, anti-nucleosome antibodies. However, only a few studies have been conducted on other autoantibodies, and the results are inconsistent. Furthermore, little is known regarding the diagnostic role of these autoantibodies in patients with new-onset SLE.

This research aims at exploring the relationship between SLE-related autoantibodies, including anti-dsDNA, anti-Sm, anti-ribosomal P protein (anti-P), anti-chromatin, anti-SSA/Ro60 (anti-Ro60), anti-SSA/Ro52 (anti-Ro52), anti-SSB, anti-centromere and anti-SmRNP antibodies, and clinicopathological features such as sex, age, disease activity, serological results and clinical manifestations in Chinese patients with new-onset SLE by retrospective cohort study.

## Methods

### Study population

According to Rao’s study^7^, to achieve clinically significant difference of clinical features and laboratory data between groups , a minimum sample size of 120 gives adequate study power to detect differences between groups (α = 0.05, power = 80%, two tailed test). A total of 526 new-onset SLE patients who met the 1997 updated ACR SLE classification criteria were enrolled between 2012 and 2021 from the First Affiliated Hospital of Bengbu Medical College after giving verbal informed consent. The basic information, clinical manifestations and serological results were collected during hospitalization. Disease activity was measured by the SLEDAI-2000 criteria. Ninety-one patients (17.3%) had late-onset SLE with age of diagnosis ≥ 50 years^8^.

The study had received ethical approval by the Ethics Committee of the First Affiliated Hospital of Bengbu Medical College [No. 2022 (149)].

### Clinical symptoms and complications

According to 1997 update ACR criteria^4^, we collected the information of the following symptoms. Facial rash includes malar rash and discoid rash. The clinical feature of arthritis is tenderness, swelling, or effusion of 2 or more peripheral joints. The diagnosis of these symptoms and oral ulcerations (oral or nasal ulcerations) mainly depends on physical exam and case history. The symptoms of neuropsychiatric systemic lupus erythematosus (NPSLE) are seizures or psychosis (exclude drug or known metabolic derangements). Diagnosis can be aided by a combination of clinical manifestations, as well as and CT and MRI. Those with persistent proteinuria > 0.5 g or 3 + or cellular casts are diagnosed with renal disorder. The main diagnostic method of serositis (including pleuritis and percarditis) is imaging examination including ultrasonic examination and CT.

Furthermore, we registered other common or special symptoms and complications^9^. Patients with fever (excluding infection), alopecia, Reynold’s phenomenon and appendicular rash can be diagnosed by physical exam and case history. The diagnosis of interstitial lung disease (ILD) and pulmonary arterial hypertension (PAH) depends on HRCT and echocardiography, respectively.

### Immunological tests

ANA levels in 475 patients were tested by immunofluorescence assay (EUROIMMUN, China), and in 22 patients by ELISA (KHB, Shanghai, China). Other autoantibodies were detected by line immunoassay (BioPlex 2200, Bio-Rad).

### Statistical analysis

Statistical analysis was performed using SPSS 16.0. Continuous variables were described as mean ± standard deviation. Using the Kolmogorov–Smirnov test to verify normal distribution of variables. The links between sex, age at diagnosis, clinical manifestations and autoantibodies were determined by the Chi-square test. The Wilcoxon rank-sum test was used to compare the laboratory measurements and disease activity between patients positive and negative for the autoantibodies. The correlation between the different autoantibodies were detected using Cluster analysis with Ward’s method. A value of *P* < 0.05 was considered significant for the above-mentioned tests.

### Ethical approval and consent to participate

Informed consent was obtained from all subjects and their legal guardians. The study had received ethical approval by the Ethics Committee of the First Affiliated Hospital of Bengbu Medical College. All methods were carried out in accordance with relevant guidelines and regulations.

## Results

### Characteristics of study subjects

The demographic characteristics, disease activity and clinical manifestations of 526 new-onset SLE patients are summarized in Table 1. The median age of patients at diagnosis was 36.52 ± 14.31 years, and 90.5% of the patients were women. The majority of patients presented medium to high disease activity (> 70%), and more than half of the patients had fever (50.2%). In addition, the common initial symptom were arthritis (48.7%), facial rash (34.0%), renal disorder (33.5%), serositis (19.2%), appendicular rash (18.1%), Reynold’s phenomenon (17.7%), alopecia (16.0%), and ILD (11.2%). In contrast, PAH, oral ulcerations and NPSLE were less than 8% frequent.

**Table 1:** Demographic characteristics and clinical manifestations of 526 Chinese patients.

Characteristics	Total (n = 526)
Female, n (%)	476 (90.5)
Age at onset, yrs, mean ± SD	35.78 ± 14.13
Age at diagnosis, yrs, mean ± SD	36.52 ± 14.31
Course of disease, mths, mean ± SD	10.92 ± 28.20
SLEDAI score	
0–6, n (%)	143 (27.2)
7–12, n (%)	183 (34.8)
≥ 13, n (%)	200 (38.0)
Clinical manifestations, n (%)	
Fever	264 (50.2)
Facial rash	179 (34.0)
Oral ulcerations	35 (6.7)
Alopecia	84 (16.0)
Reynold’s phenomenon	93 (17.7)
Neuropsychiatric systemic lupus erythematosus (NPSLE)	16 (3.0)
Serositis	101 (19.2)
Renal disorder	176 (33.5)
Appendicular rash	95 (18.1)
Pulmonary arterial hypertension (PAH)	39 (7.4)
Interstitial lung disease (ILD)	59 (11.2)
Arthritis	256 (48.7)

### Prevalence of autoantibodies

As shown in Table 2, 97.4% of 497 patients tested positive for the anti-ANA antibody. In addition, the anti-chromatin antibody also had a high positive rate of 72.6%, followed by the anti-dsDNA (69%), anti-Ro60 (66%), anti-SmRNP (62.4%), anti-Sm (56.3%), anti-Ro52 (51.5%), anti-P (39.4%) and anti-SSB (28.7%) antibodies. In contrast, less than 5% of the patients were positive for the anti-centromere antibody.

**Table 2:** Positive rate of autoantibodies of 526 patients.

	n (%)
ANA	484/497 (97.4)
Anti-dsDNA	363 (69.0)
Anti-chromatin	382 (72.6)
Anti-P	207 (39.4)
Anti-Sm	296 (56.3)
Anti-SmRNP	328 (62.4)
Anti-Ro60	347 (66.0)
Anti-Ro52	271 (51.5)
Anti-SSB	151 (28.7)
Anti-centromere	20 (3.8)

### The association of autoantibodies with sex and age

The relationship between sex, age at onset and autoantibodies is shown in Table 3. The prevalence of anti-P antibody was significantly higher in female patients compared to that in male patients (41.2% vs 22.0%, *P* = 0.008). Furthermore, the positive rate of anti-Sm antibody in early-onset patients was 58.9% compared to 44% in the late-onset patients (*P* = 0.009).

**Table 3:** Sex and age at onset in relation to autoantibodies.

	Sex		*P*	Age at onset		*P*
	Male, n (%)	Female, n (%)		< 50 years, n (%)	≥ 50 years, n (%)	
dsDNA (+)	32 (64.0)	331 (69.5)	0.421	308 (70.8)	55 (60.4)	0.052
Sm (+)	29 (58.0)	267 (56.1)	0.796	256 (58.9)	40 (44.0)	*0.009*
P (+)	11 (22.0)	196 (41.2)	*0.008*	178 (40.9)	29 (31.9)	0.108
Ro60 (+)	33 (66.0)	314 (66.0)	0.996	290 (66.7)	57 (62.6)	0.461
Ro52 (+)	24 (48.0)	247 (51.9)	0.601	230 (52.9)	41 (45.1)	0.175
SSB (+)	15 (3.0)	136 (28.6)	0.832	130 (29.9)	21 (23.1)	0.192
Centromere (+)	39 (78.0)	343 (72.1)	0.370	321 (73.8)	61 (67.0)	0.188
SmRNP (+)	33 (66.0)	295 (62.0)	0.576	276 (63.4)	52 (57.1)	0.259

Significant values are in [italics].

### The association of autoantibodies with disease activity and laboratory data

The relationship between disease activity, laboratory data and autoantibodies is shown in Table 4. The SLEDAI score was significantly higher in patients with the anti-dsDNA (*P* < 0.001), anti-P (*P* = 0.042) and anti-chromatin (*P* = 0.001) antibodies compared to patients lacking these autoantibodies. In addition, the prevalence of anti-dsDNA (*P* < 0.001), anti-P (*P* < 0.001), anti-Ro60 (*P* = 0.009), anti-Ro52 (*P* = 0.014) and anti-SSB (*P* = 0.004) antibodies could result in a significantly rapider erythrocyte sedimentation rate (ESR). The presence of anti-Sm (*P* = 0.001), anti-Ro60 (*P* < 0.001), anti-Ro52 (*P* < 0.001), anti-SSB (*P* < 0.001) and anti-SmRNP (*P* = 0.001) antibodies correlated with higher levels of Immunoglobulin G (IgG), whereas patients with anti-SSB antibodies (*P* = 0.006) had lower concentrations of Immunoglobulin A (IgA). Complement C_3_ and C_4_ were significantly lower in patients with anti-dsDNA (*P* < 0.001), anti-P (*P* = 0.003; *P* = 0.001 respectively), anti-SSB (*P* < 0.001; *P* = 0.001, respectively) and anti-chromatin (*P* = 0.001; *P* = 0.023, respectively) antibodies, and patients with anti-centromere antibodies (*P* = 0.041) had lower levels of complement C_4_. Furthermore, subjects with anti-dsDNA (*P* < 0.001), anti-Ro60 (*P* = 0.002), anti-Ro52 (*P* = 0.003) and anti-SSB (*P* < 0.001; *P* = 0.013, respectively) antibodies had lower levels of hemoglobin (Hb) and lower white blood cell (WBC) counts. The anti-SmRNP antibodies (*P* = 0.021) correlated with higher Hb levels, and the anti-centromere antibodies (*P* = 0.039) with lower WBC counts. While anti-dsDNA (*P* = 0.038) and anti-SSB (*P* = 0.031) antibodies correlated significantly with lower platelet (Plt) count, patients with anti-SmRNP antibodies (*P* = 0.011) had a higher Plt count. Patients with anti-SSB (*P* = 0.004; *P* = 0.008, respectively) antibodies had higher levels of alanine aminotransferase (ALT) and aspartate transaminase (AST), but patients with anti-P (*P* = 0.014; *P* = 0.047, respectively) had a lower levels of ALT and AST. Moreover, patients with anti-Sm antibody (*P* = 0.022) had a higher levels of AST, whereas the presence of anti-SmRNP antibody (*P* = 0.032) was associated with lower AST levels. The prevalence of anti-dsDNA (*P* < 0.001), anti-Ro60 (*P* = 0.020) and anti-SSB (*P* = 0.021) antibodies were related to lower albumin (Alb) concentrations. Finally, anti-dsDNA (*P* = 0.049; *P* = 0.037 respectively) correlated significantly with higher levels of serum creatinine (sCr) and blood urea nitrogen (BUN), whereas the anti-P antibody (*P* = 0.035; *P* = 0.008, respectively) correlated with lower levels of these indicators.

**Table 4:** Association between disease activity, laboratory data and autoantibodies.

		SLEDAI score	*P*	ESR	*P*	IgG	*P*	IgA	*P*	C_3_	*P*	C_4_	*P*	Hb	*P*	WBC	*P*	Plt	*P*	AST	*P*	ALT	*P*	ALB	*P*	sCr	*P*	BUN	*P*
dsDNA	+	12.38 ± 5.69	< *0.001*	58.79 ± 31.14	< *0.001*	20.45 ± 7.99	0.996	3.08 ± 1.41	0.883	0.43 ± 0.21	< *0.001*	0.07 ± 0.06	< *0.001*	98.45 ± 20.35	< *0.001*	4.37 ± 2.65	< *0.001*	167.93 ± 90.13	*0.038*	56.75 ± 104.64	0.858	40.10 ± 70.75	0.855	32.64 ± 6.83	< *0.001*	66.19 ± 64.06	*0.049*	5.61 ± 3.83	*0.037*
	−	7.70 ± 4.75		46.26 ± 28.44		20.99 ± 10.05		3.09 ± 0.1.64		0.64 ± 0.21		0.13 ± 0.06		107.78 ± 20.44		5.35 ± 2.84		184.07 ± 91.08		59.44 ± 138.36		45.44 ± 83.53		36.03 ± 6.63		58.19 ± 37.15		5.08 ± 3.29	
Sm	+	11.02 ± 5.88	0.767	55.53 ± 30.96	0.624	21.43 ± 8.14	*0.001*	3.03 ± 1.30	0.854	0.49 ± 0.24	0.699	0.09 ± 0.07	0.274	102.62 ± 20.82	0.168	4.60 ± 2.68	0.454	174.58 ± 87.38	0.354	58.48 ± 119.45	*0.022*	39.26 ± 61.52	0.306	33.79 ± 7.08	0.707	65.23 ± 69.61	0.138	5.33 ± 3.57	0.165
	−	10.82 ± 5.78		53.95 ± 30.73		19.58 ± 9.22		3.15 ± 1.69		0.49 ± 0.22		0.09 ± 0.06		99.74 ± 20.74		4.78 ± 2.83		170.88 ± 94.95		56.45 ± 111.97		45.00 ± 89.29		33.59 ± 6.78		61.65 ± 34.78		5.59 ± 3.79	
*P*	+	11.51 ± 5.63	*0.042*	60.55 ± 28.24	< *0.001*	20.58 ± 7.51	0.639	3.04 ± 1.32	0.866	0.45 ± 0.21	*0.003*	0.08 ± 0.06	*0.001*	101.00 ± 20.03	0.674	4.37 ± 2.47	0.071	172.98 ± 90.65	0.89	38.51 ± 48.19	*0.047*	28.90 ± 25.70	*0.014*	34.27 ± 6.38	0.16	58.35 ± 25.86	*0.035*	4.97 ± 2.93	*0.008*
	−	10.55 ± 5.93		51.26 ± 31.90		20.63 ± 9.37		3.11 ± 1.58		0.52 ± 0.24		0.10 ± 0.07		101.60 ± 21.33		4.87 ± 2.89		173.28 ± 93.18		69.95 ± 142.67		50.08 ± 93.00		33.35 ± 7.27		67.15 ± 70.18		5.75 ± 4.06	
R60	+	11.08 ± 5.75	0.308	57.54 ± 31.35	*0.009*	21.47 ± 8.55	< *0.001*	3.13 ± 1.54	0.353	0.48 ± 0.24	0.136	0.09 ± 0.07	0.943	99.51 ± 20.63	*0.002*	4.47 ± 2.75	*0.002*	166.80 ± 87.18	0.056	58.16 ± 117.69	0.477	42.40 ± 73.02	0.166	33.20 ± 6.83	*0.02*	62.14 ± 44.79	0.597	5.35 ± 3.60	0.953
	−	10.64 ± 5.99		49.71 ± 29.26		18.95 ± 8.70		2.98 ± 1.37		0.51 ± 0.23		0.09 ± 0.07		104.94 ± 20.76		5.07 ± 2.70		184.85 ± 96.12		56.52 ± 113.42		40.61 ± 78.69		34.67 ± 7.07		66.53 ± 74.91		5.61 ± 3.81	
R52	+	11.10 ± 5.68	0.393	58.55 ± 32.23	*0.014*	22.46 ± 9.02	< *0.001*	2.98 ± 1.48	0.095	0.47 ± 0.22	0.157	0.09 ± 0.07	0.148	98.94 ± 20.47	*0.003*	4.41 ± 2.74	*0.003*	170.98 ± 87.18	0.643	55.29 ± 96.71	0.299	42.09 ± 76.75	0.944	33.36 ± 6.66	0.218	63.26 ± 49.01	0.87	5.41 ± 3.80	0.977
	−	10.75 ± 5.99		50.88 ± 28.82		18.63 ± 7.83		3.19 ± 1.48		0.51 ± 0.24		0.09 ± 0.07		103.93 ± 20.91		4.96 ± 2.73		175.10 ± 94.31		60.04 ± 133.90		41.45 ± 73.27		34.06 ± 7.22		64.06 ± 64.49		5.48 ± 3.53	
SSB	+	11.70 ± 6.07	0.074	61.61 ± 31.92	*0.004*	23.40 ± 9.36	< *0.001*	2.90 ± 1.66	*0.006*	0.42 ± 0.22	< *0.001*	0.07 ± 0.06	*0.001*	95.31 ± 20.49	< *0.001*	4.30 ± 2.58	*0.013*	159.88 ± 91.06	*0.031*	63.93 ± 103.56	*0.008*	43.95 ± 73.12	*0.004*	32.67 ± 7.05	*0.021*	67.29 ± 61.06	0.082	5.38 ± 3.24	0.463
	−	10.62 ± 5.71		52.15 ± 30.03		19.47 ± 8.12		3.15 ± 1.40		0.52 ± 0.23		0.10 ± 0.07		103.78 ± 20.47		4.83 ± 2.80		178.18 ± 90.09		55.06 ± 120.84		40.92 ± 75.75		34.12 ± 6.86		62.64 ± 55.40		5.46 ± 3.83	
Centromere	+	11.95 ± 6.59	0.538	60.28 ± 31.96	0.404	23.88 ± 12.35	0.213	3.73 ± 2.01	0.148	0.40 ± 0.19	0.164	0.06 ± 0.04	*0.041*	94.80 ± 17.29	0.112	3.36 ± 1.35	*0.039*	145.35 ± 114.92	0.087	41.00 ± 38.41	0.79	30.75 ± 25.17	0.501	30.86 ± 6.92	0.084	87.5 ± 130.48	0.98	7.23 ± 7.35	0.821
	−	10.90 ± 5.80		54.68 ± 30.83		20.48 ± 8.50		3.06 ± 1.46		0.49 ± 0.23		0.09 ± 0.07		101.56 ± 20.88		4.73 ± 2.78		174.07 ± 89.59		57.80 ± 117.84		41.03 ± 71.55		33.82 ± 6.93		62.81 ± 52.60		5.38 ± 3.46	
Chromatin	+	11.43 ± 5.89	*0.001*	55.13 ± 30.83	0.781	20.67 ± 8.02	0.262	3.10 ± 1.43	0.381	0.47 ± 0.23	*0.001*	0.09 ± 0.07	*0.023*	101.09 ± 20.95	0.564	4.52 ± 2.65	0.058	172.90 ± 88.09	0.827	54.02 ± 104.55	0.073	39.89 ± 76.79	0.138	33.36 ± 6.90	0.1	65.33 ± 62.32	0.095	5.47 ± 3.34	0.071
	−	9.60 ± 5.47		54.11 ± 30.97		20.46 ± 10.23		3.03 ± 1.62		0.54 ± 0.22		0.10 ± 0.07		102.09 ± 20.50		5.10 ± 2.95		173.21 ± 97.43		67.13 ± 142.52		46.81 ± 69.85		34.65 ± 6.99		59.22 ± 39.48		5.36 ± 4.45	
SmRNP	+	11.20 ± 5.89	0.223	55.66 ± 30.79	0.456	21.32 ± 8.10	*0.001*	3.06 ± 1.33	0.542	0.50 ± 0.24	0.323	0.09 ± 0.07	0.814	103.07 ± 20.74	*0.021*	4.61 ± 2.62	0.774	178.70 ± 86.00	*0.011*	56.79 ± 114.63	*0.032*	39.80 ± 67.68	0.578	33.97 ± 7.04	0.217	65.29 ± 66.77	0.461	5.34 ± 3.57	0.106
	−	10.49 ± 5.71		53.51 ± 30.97		19.45 ± 9.56		3.13 ± 1.71		0.47 ± 0.22		0.09 ± 0.07		98.52 ± 20.68		4.78 ± 2.94		163.39 ± 97.43		58.92 ± 118.85		45.03 ± 85.67		33.27 ± 6.77		60.97 ± 35.49		5.60 ± 3.83	

Significant values are in [italics]

### The association of autoantibodies with clinical manifestations

The correlation between clinical manifestations and the different autoantibodies are summarized in Table 5. Fever was significantly associated with the presence of anti-dsDNA (*P* = 0.009), anti-P (*P* = 0.031) and anti-SmRNP (*P* = 0.041) antibodies. Facial rash was more frequent in patients positive for anti-dsDNA (*P* = 0.013), anti-P (*P* < 0.001) and anti-SmRNP (*P* = 0.031) antibodies, whereas patients with anti-R52 antibody (*P* = 0.039) were less likely to develop facial rash. Alopecia was associated with anti-dsDNA (*P* = 0.020), anti-Sm (*P* = 0.020) and anti-SmRNP (*P* = 0.034) antibodies, Reynold’s phenomenon with anti-Sm (*P* = 0.007) and anti-SmRNP (*P* < 0.001) antibodies, and serositis with anti-dsDNA (*P* = 0.014), anti-Ro52 (*P* = 0.047) and anti-centromere (*P* = 0.016) antibodies. While the presence of anti-P (*P* = 0.003) and anti-SmRNP (*P* = 0.001) antibodies correlated with a higher risk of appendicular rash, patients with anti-SSB antibody (*P* = 0.038) were less likely to develop the same. PAH was associated significantly with the presence of anti-dsDNA antibody (*P* = 0.021), whereas the anti-P antibody (*P* = 0.009; *P* = 0.012, respectively) was correlated with a lower risk of PAH and ILD. In addition, the patients with anti-dsDNA (*P* = 0.013) and anti-R60 (*P* = 0.044) antibodies were less likely to develop ILD. Renal disorder was associated with the presence of anti-dsDNA (*P* < *0.001*) and anti-SSB (*P* = *0.006*) antibodies. Anti-SmRNP antibody was also correlated with oral ulcerations (*P* = 0.026), whereas patients with anti-SmRNP antibody (*P* = 0.037) were less likely to develop NPSLE. There was no relationship between the different autoantibodies and arthritis.

**Table 5:** Correlation between clinical manifestations and autoantibodies.

	dsDNA (+)	*P*	Sm (+)	*P*	P (+)	*P*	R60 (+)	*P*	R52 (+)	*P*	SSB (+)	*P*	Centromere ( +)	*P*	SmRNP ( +)	*P*
Patients number	363		296		207		347		271		151		20		328	
Fever (n = 264)	196	*0.009*	156	0.191	116	*0.031*	184	0.07	135	0.859	83	0.164	8	0.348	176	*0.041*
Facial rash (n = 179)	136	*0.013*	107	0.245	97	< *0.001*	119	0.859	81	*0.039*	59	0.121	5	0.382	123	*0.031*
Oral ulcerations (n = 35)	24	0.954	21	0.646	12	0.525	25	0.481	21	0.299	8	0.428	1	0.761	28	*0.026*
Alopecia (n = 84)	67	*0.02*	57	*0.02*	38	0.228	61	0.161	48	0.261	27	0.448	1	0.171	61	*0.034*
Reynold’s phenomenon (n = 93)	58	0.127	64	*0.007*	35	0.708	62	0.876	53	0.245	19	0.052	3	0.746	77	< *0.001*
NPSLE (n = 16)	8	0.095	6	0.124	5	0.5	11	0.812	9	0.701	7	0.177	1	0.605	6	*0.037*
Serositis (n = 101)	80	*0.014*	60	0.48	39	0.866	68	0.749	61	*0.047*	31	0.624	8	*0.016*	61	0.651
Appendicular rash (n = 95)	59	0.108	57	0.419	50	*0.003*	61	0.689	43	0.178	19	*0.038*	3	0.741	74	*0.001*
PAH (n = 39)	33	*0.021*	38	0.181	14	*0.009*	34	0.151	32	0.658	14	0.37	3	0.587	40	0.36
ILD (n = 59)	20	*0.013*	21	0.751	8	*0.012*	20	*0.044*	19	0.716	10	0.66	0	0.196	25	0.815
Arthritis (n = 256)	183	0.232	145	0.869	108	0.195	164	0.369	132	0.985	72	0.774	6	0.087	165	0.334
Renal disorder (n = 176)	140	< *0.001*	100	0.858	62	0.17	121	0.34	97	0.242	64	*0.006*	8	0.527	110	0.962

Significant values are in [italics]

### The relationship between autoantibodies

**Figure 1:**
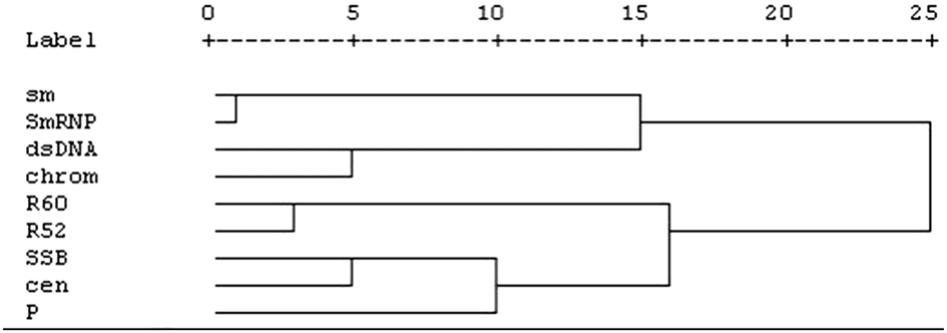
The relationship between the autoantibodies were detected by Cluster analysis. Chrom: chromatin; cen: centromere. Cluster 1 was composed of anti-Sm antibody and anti-SmRNP antibody. Cluster 2 was composed of anti-R60 antibody and anti-R52 antibody. Cluster 3 was composed of anti-dsDNA antibody and anti-chromatin antibody. Cluster 4 was composed of anti-SSB and anti-Centromere antibodies. Cluster 5 was composed of anti-P antibody.

The relationship between autoantibodies were detected by Cluster analysis. As shown in Fig. 1, the autoantibodies were classified into 5 clusters. Cluster 1 included the anti-Sm and anti-SmRNP antibodies, and Cluster 2 was composed of anti-R60 and anti-R52 antibodies. Both clusters were formed early in the disease course. Cluster 3 comprised of anti-dsDNA and anti-chromatin antibodies, Cluster 4 included the anti-SSB and anti-centromere antibodies, and Cluster 5 included the anti-P antibody.

## Discussion

SLE is characterized by the presence of specific autoantibodies^1^. The therapy which focus on autoantibodies has been cared much. For example, blocking anti-dsDNA antibodies in a mouse model of SLE alleviated organ injury^5^. In this study, we retrospectively analyzed the correlation of several autoantibodies with sex, age at onset, disease activity, laboratory data and clinical manifestations in Chinese patients with new-onset SLE, in order to identify novel therapy.

In this study, fever and arthritis are common initial symptoms, which was also observed in other study^9,10^. Arthritis were more prevalent in some studies^11,12^, which may be attributed to different definition of it. The prevalence of facial rash from our study was similar to the study by Sebastiani et al.^11^, but lower than that in some previous studies^12,13^. Meanwhile, the ocurring rate of appendicular rash which is part of skin manifestation, alopecia and Reynold’s phenomenon lower than that in other studies^10,12,13^. Skin manifestation is influenced by sun-exposing, and discoid rash can induce alopecia^9,11^. Cold stimulation is a cause of Reynold’s phenomenon. The difference may be due to geological location, climate, as well as genetic factors. NPSLE with more nonspecific symptoms like headache and mood disorder is difficult to diagnose. Therefore, the prevalence of NPSLE was different in previous cohorts, but still a rare initial presentation^11,13^. The incidence of serositis in our cohort was consistent with the finding of Leuchten et al.^13^, but lower than that in study by Sebastiani et al.^11^. The first incidence rate of serositis which diagnosis is dependent on the imaging examination may be underestimated. The frequency of renal disorder was essentially comparable with that from other researches^11,13^. We did not find the first incidence rate of PAH and ILD in previous studies, which are rare symptoms, but the outcomes were coincide with the incidence of them in the course of SLE^14^.

The prevalence of anti-P (39.4%), anti-Ro60 (66.0%) and anti-Ro52 (51.5%) antibodies were higher in our cohort compared to that reported in previous studies^15–20^, which may be attributed to different ethnicities^21^.

The anti-dsDNA antibody is a reliable diagnostic biomarker for SLE^16^, and its presence is related to tissue damage in the kidneys, skin and brain^5^. Our results just conformed to the first two. The difference may be due to different course of disease and sample size. Another possible explanation is the difficulty of diagnosis for NPSLE and the rarity of it as initial symptom. We found that the anti-dsDNA antibody was associated with disease activity, leukopenia, anemia, serositis, thrombocytopenia, ESR, complement C_4_ and Alb, which is consistent with previous findings^22–24^. Higher ESR is the likely result of kidney damage, which in turn lowers Alb levels^25^. Furthermore, presence of the anti-dsDNA antibody was related to more serious kidney injury, lower levels of complement C_3_, fever, alopecia, PAH and less prevalence of ILD. According to the research by Li et al.^26^, the frequency of lupus nephritis, serositis and hypocomplementemia in patients with SLE and PAH were significant higher. Meanwhile, the disease activity of them were higher, and our results of association of anti-dsDNA were coincide with above symptoms. PAH and ILD were rare initial sign. We will collect more data of new-onset SLE paitents with them, and conduct follow-up studies in risk factors of these complications in the future.

The anti-Sm antibody is highly specific for SLE, and we detected significantly higher positive rate of this autoantibody in patients with early-onset SLE compared to those with late-onset disease. Previous studies have shown that SLE patients with anti-Sm antibody tend to be younger compared to those lacking the antibody^17,27^. In addition, the anti-Sm antibody was associated with Reynold’s phenomenon and elevated liver enzymes in our study, as demonstrated by other groups as well^24,28^. Alopecia and higher levels of IgG also correlated with the presence of anti-Sm antibody in our cohort. There is evidence that anti-Sm antibody is associated with disease activity^17,29^, renal disorder^28,30,31^ and lower levels of complement proteins^31,32^. The differences in the testing methods for anti-Sm antibody may explain the variations in results^33^.

The anti-P antibody has been previously associated with disease activity^18,34^, lower levels of complement^34^, fever^18,21^ and malar rash^18,35,36^ in SLE patients, which was also observed in our study. However, our findings contradict the previously reported correlation between anti-P antibody and renal disorder^21,36,37^. In fact, we found that the extent of kidney damage of patients with anti-P antibody was slighter than that without it. Furthermore, the liver function of patients with anti-P antibody was better compared to those lacking the antibody, which is also inconsistent with previous studies^21,38^. In a previous study^39^, the levels of anti- P antibody increased during the active phase of nephritis and resumed to normal in remission stage. In addition, the patient with anti-P antibody was not diagnosed with liver damage at early stage of SLE. Therefore, these discrepancies can be attributed to differences in course of disease, meanwhile, it also may be associated with ethnicity and study design. More follow-up is warranted to observe the long-term complications. Moreover, the presence of anti-P antibody was correlated to ESR, appendicular rash, and lower prevalence of PAH and ILD in our study. However, we did not observe any correlation between anti-P antibody and NPSLE which is difficult to diagnose. One noteworthy finding was that the prevalence of anti-P antibody was higher in female patients than in male patients.

The prevalence of anti-SSA and anti-SSB antibodies was higher in SLE patients with secondary Sjogren’s syndrome. Earlier studies have demonstrated that anti-SSA (Ro) antibody is related to hemocytopenia^40^ and ILD^41^. Our findings regarding the former were similar, whereas that regarding ILD were contradictory, which can be attributed to differences in sample size and ethnic groups. Moreover, ILD mostly occurred in long-course patients^14^, different course of disease may explain the discrepancy. We also found that anti-SSA antibody was correlated to ESR and higher levels of IgG. In addition, we observed a correlation between anti-Ro60 antibody and lower Alb concentrations. The presence of the anti-Ro52 antibody was related to lower prevalence of malar rash, which contradicts the findings of Harley et al.,^40^ which can be explained by different definition. Some studies have reported an association between anti-SSA antibody and PAH^26,42^, and the risk factors of PAH are pericarditis and pleurisy^26^, which were not observed in our study. However, we detected a correlation between the anti-Ro52 antibody and serositis, which has been reported previously^24^. There may be a potential link between anti-SSA and PAH, and further studies are needed to verify this hypothesis. Previous studies have shown that anti-SSB antibody is associated with higher levels of IgG^7^, lower levels of Complement C_3_^23^ and hematological symptoms^7,42^, which was confirmed in our study as well. In addition, we found that anti-SSB antibody correlated to ESR, lower levels of IgA and Complement C_4_, lower Alb concentrations, lower prevalence of appendicular rash, higher prevalence of renal disorder and more serious hepatic damage. We observed that the patients with anti-SSB antibody got lower levels of Hb, WBC and Plt which is initial hematological symptoms. It may be caused by disease activity, renal disorder or liver damage, and this new result need further follow-up observation.

The anti-centromere antibody (ACA) has been detected in subjects with CREST syndrome. The positivity rate of ACA in SLE patients in our study was 3.8%. In addition, ACA was correlated to lower levels of Complement C_4_, lower WBC count and serositis. However, we did not detect any association between ACA and Reynold’s phenomenon, most likely due to the few samples positive for ACA. The prevalence of anti-chromatin antibody (also called anti-nucleosome antibody) is high in SLE patients^20^, and is associated with disease activity and renal disorder^20,43,44^. We also detected an association between this antibody and disease activity but without renal disorder, which may be due to genetic, course of disease and ethnic influences. Furthermore, the anti-chromatin antibody was correlated to hypo-complementemia in our study. The anti-SmRNP antibody is derived from anti-RNP antibody^45^, and is associated with higher levels of IgG and Hb, higher Plt count, lower levels of AST, fever, skin manifestations, oral ulcerations, Reynold’s phenomenon, alopecia and lower prevalence of NPSLE. There is some overlap between the clinical manifestations of anti-SmRNP and anti-RNP antibodies, such as Reynold’s phenomenon^46^. Using cluster analysis, the autoantibodies were classified into 5 clusters. Only cluster 1 and cluster 5 fit well with previous studies^42,47^, which could be explained by the different autoantibodies we detected. In addition, anti-dsDNA antibody was highly relevant with anti-chromatin antibody in previous study, which was observed in our study (cluster 3)^44^.

Our study has certain salient features, such as a large cohort and all patients with new-onset SLE. Thus, our findings are more relevant in terms of identifying targets for delaying the progression of SLE, since because the prevalence of autoantibodies can change during disease course^48^. Nevertheless, we could not examine the changes in the spectrum and levels autoantibodies during the course of SLE due to the cross-sectional design of our study. Second, our cohort consisted of only Chinese patients, and the results may not be applicable to other ethnic populations. Third, samples with rare autoantibodies and clinical manifestations were few, and the findings will have to be validated with further studies.

In conclusion, detection of specific autoantibodies in SLE patients can predict organ injury and other complications, and aid in timely intervention. We recommend that patients with anti-dsDNA antibodies should undergo echocardiography to detect PAH in a timely manner, and liver function tests should be conducted for those with anti-SSB antibody. The blood routine examination need to be tested regularly for patients with anti-SSA, anti-SSB and anti-centromere antibodies. Patients with anti- dsDNA and anti-SSB antibody should pay attention to tests of renal function. Imaging examination of such as echocardiography and CT need to be performed to detect serositis for patients with anti-dsDNA, anti-Ro52 and anti-centromere antibodies.

## Data Availability

The datasets used and/or analyzed during this study are available from the corresponding author on reasonable request.

## References

[1] Olsen NJ, Karp DR (2014). Autoantibodies and SLE: The threshold for disease. Nat. Rev. Rheumatol..

[2] Xiao ZX, Miller JS, Zheng SG (2021). An updated advance of autoantibodies in autoimmune diseases. Autoimmun Rev..

[3] Fava A, Petri M (2019). Systemic lupus erythematosus: Diagnosis and clinical management. J. Autoimmun..

[4] Hochberg MC (1997). Updating the American College of Rheumatology revised criteria for the classification of systemic lupus erythematosus. Arthritis Rheum..

[5] Wang X, Xia Y (2019). Anti-double stranded DNA antibodies: Origin, pathogenicity, and targeted therapies. Front. Immunol..

[6] Sulcebe G, Morcka K (1992). Diagnostic and prognostic significance of different antinuclear antibodies in more than 1000 consecutive Albanian patients with rheumatic diseases. Clin. Exp. Rheumatol..

[7] Rao L, Liu G, Li C, Li Y, Wang Z, Zhou Z, Tong S, Wu X (2013). Specificity of anti-SSB as a diagnostic marker for the classification of systemic lupus erythematosus. Exp. Ther. Med..

[8] Arnaud L, Mathian A, Boddaert J, Amoura Z (2012). Late-onset systemic lupus erythematosus: Epidemiology, diagnosis and treatment. Drugs Aging.

[9] Kuhn A, Bonsmann G, Anders HJ, Herzer P, Tenbrock K, Schneider M (2015). The diagnosis and treatment of systemic lupus erythematosus. Deutsches Arzteblatt Int..

[10] Metry AM, Al Salmi I, Al Balushi F, Yousef MA, Al Ismaili F, Hola A, Hannawi S (2019). Systemic lupus erythematosus: Symptoms and signs at initial presentations. AntiInflamm. Antiallergy Agents Med. Chem..

[11] Sebastiani GD, Prevete I, Iuliano A, Minisola G (2016). The importance of an early diagnosis in systemic lupus erythematosus. Isr. Med. Assoc. J. IMAJ.

[12] Nossent J, Kiss E, Rozman B, Pokorny G, Vlachoyiannopoulos P, Olesinska M, Marchesoni A, Mosca M, Påi S, Manger K, Schneider M, Nielsen H, van Vollenhoven R, Swaak T (2010). Disease activity and damage accrual during the early disease course in a multinational inception cohort of patients with systemic lupus erythematosus. Lupus.

[13] Leuchten N, Milke B, Winkler-Rohlfing B, Daikh D, Dörner T, Johnson SR, Aringer M, on behalf of the SLE Classification Criteria Steering Committee (2018). Early symptoms of systemic lupus erythematosus (SLE) recalled by 339 SLE patients. Lupus.

[14] Hannah JR, D'Cruz DP (2019). Pulmonary complications of systemic lupus erythematosus. Sem. Respir. Crit. Care Med..

[15] Pisetsky DS, Bossuyt X, Meroni PL (2019). ANA as an entry criterion for the classification of SLE. Autoimmun. Rev..

[16] Dema B, Charles N (2016). Autoantibodies in SLE: Specificities, isotypes and receptors. Antibodies (Basel Switz.).

[17] Flechsig A, Rose T, Barkhudarova F, Strauss R, Klotsche J, Dähnrich C, Schlumberger W, Enghard P, Burmester GR, Hiepe F, Biesen R (2017). What is the clinical significance of anti-Sm antibodies in systemic lupus erythematosus? A comparison with anti-dsDNA antibodies and C3. Clin. Exp. Rheumatol..

[18] Abraham M, Derk CT (2015). Anti-ribosomal-P antibodies in lupus nephritis, neuropsychiatric lupus, lupus hepatitis, and Chagas’ disease: Promising yet limited in clinical utility. Rheumatol. Int..

[19] Didier K, Bolko L, Giusti D, Toquet S, Robbins A, Antonicelli F, Servettaz A (2018). Autoantibodies associated with connective tissue diseases: What meaning for clinicians?. Front. Immunol..

[20] Burlingame RW, Cervera R (2002). Anti-chromatin (anti-nucleosome) autoantibodies. Autoimmun. Rev..

[21] Pasoto SG, Viana VS, Bonfa E (2014). The clinical utility of anti-ribosomal P autoantibodies in systemic lupus erythematosus. Expert Rev. Clin. Immunol..

[22] Conti F, Ceccarelli F, Perricone C, Massaro L, Marocchi E, Miranda F, Spinelli FR, Truglia S, Alessandri C, Valesini G (2015). Systemic Lupus Erythematosus with and without Anti-dsDNA antibodies: Analysis from a large monocentric cohort. Med. Inflamm..

[23] Correa-Rodríguez M, Pocovi-Gerardino G, Callejas-Rubio JL, Ríos-Fernández R, Martín-Amada M, Cruz-Caparrós MG, Rueda-Medina B, Ortego-Centeno N (2021). Clinical and serological associations of autoantibodies in patients with systemic lupus erythematosus. J. Investig. Med. Off. Publ. Am. Fed. Clin. Res..

[24] Vilá LM, Molina MJ, Mayor AM, Peredo RA, Santaella ML, Vilá S (2006). Clinical and prognostic value of autoantibodies in puerto Ricans with systemic lupus erythematosus. Lupus.

[25] Aringer M (2020). Inflammatory markers in systemic lupus erythematosus. J. Autoimmun..

[26] Li M, Wang Q, Zhao J, Li Z, Ye Z, Li C, Li X, Zhu P, Wang Z, Zheng Y, Li X, Zhang M, Tian Z, Liu Y, He J, Zhang F, Zhao Y, Zeng X, CSTAR co-authors,  (2014). Chinese SLE treatment and research group (CSTAR) registry: II. Prevalence and risk factors of pulmonary arterial hypertension in Chinese patients with systemic lupus erythematosus. Lupus.

[27] López P, Mozo L, Gutiérrez C, Suárez A (2003). Epidemiology of systemic lupus erythematosus in a northern Spanish population: Gender and age influence on immunological features. Lupus.

[28] Arroyo-Ávila M, Santiago-Casas Y, McGwin G, Cantor RS, Petri M, Ramsey-Goldman R, Reveille JD, Kimberly RP, Alarcón GS, Vilá LM, Brown EE (2015). Clinical associations of anti-Smith antibodies in PROFILE: A multi-ethnic lupus cohort. Clin. Rheumatol..

[29] Ahn SS, Jung SM, Yoo J, Lee SW, Song JJ, Park YB (2019). Anti-Smith antibody is associated with disease activity in patients with new-onset systemic lupus erythematosus. Rheumatol. Int..

[30] Alba P, Bento L, Cuadrado MJ, Karim Y, Tungekar MF, Abbs I, Khamashta MA, D'Cruz D, Hughes GR (2003). Anti-dsDNA, anti-Sm antibodies, and the lupus anticoagulant: Significant factors associated with lupus nephritis. Ann. Rheum. Dis..

[31] Ni JD, Yao X, Pan HF, Li XP, Xu JH, Ye DQ (2009). Clinical and serological correlates of anti-Sm autoantibodies in Chinese patients with systemic lupus erythematosus: 1,584 cases. Rheumatol. Int..

[32] Singh RR, Malaviya AN, Kailash S, Varghese T (1991). Clinical significance of anti-Sm antibody in systemic lupus erythematosus & related disorders. Indian J. Med. Res..

[33] López-Longo FJ, González Fernández CM, Rodríguez Mahou M, Grau Simó R, Monteagudo Sáez I, Meno García AC, Carreño Pérez L (1997). Expresión clínica del lupus eritematoso sistémico con anticuerpos anti-U1-RNP y anti-Sm [Clinical expression of systemic lupus erythematosus with anti-U1-RNP and anti-Sm antibodies]. Rev. Clin. Esp..

[34] Mahler M, Agmon-Levin N, van Liempt M, Shoenfeld Y, Waka A, Hiepe F, Swart A, Gürtler I, Fritzler MJ (2012). Multi-center evaluation of autoantibodies to the major ribosomal P C22 epitope. Rheumatol. Int..

[35] Briani C, Lucchetta M, Ghirardello A, Toffanin E, Zampieri S, Ruggero S, Scarlato M, Quattrini A, Bassi N, Ermani M, Battistin L, Doria A (2009). Neurolupus is associated with anti-ribosomal P protein antibodies: An inception cohort study. J. Autoimmun..

[36] Mahler M, Kessenbrock K, Szmyrka M, Takasaki Y, Garcia-De La Torre I, Shoenfeld Y, Hiepe F, Shun-le C, von Mühlen CA, Locht H, Höpfl P, Wiik A, Reeves W, Fritzler MJ (2006). International multicenter evaluation of autoantibodies to ribosomal P proteins. Clin. Vaccine Immunol. CVI.

[37] Gerli R, Caponi L (2005). Anti-ribosomal P protein antibodies. Autoimmunity.

[38] Kiss E, Shoenfeld Y (2007). Are anti-ribosomal P protein antibodies relevant in systemic lupus erythematosus?. Clin. Rev. Allergy Immunol..

[39] Reichlin M (2006). Autoantibodies to the ribosomal P proteins in systemic lupus erythematosus. Clin. Exp. Med..

[40] Harley JB, Scofield RH, Reichlin M (1992). Anti-Ro in Sjögren’s syndrome and systemic lupus erythematosus. Rheumatic diseases clinics of North America.

[41] Boulware DW, Hedgpeth MT (1989). Lupus pneumonitis and anti-SSA (Ro) antibodies. J. Rheumatol..

[42] Li J, Leng X, Li Z, Ye Z, Li C, Li X, Zhu P, Wang Z, Zheng Y, Li X, Zhang M, Tian XP, Li M, Zhao J, Zhang FC, Zhao Y, Zeng X (2014). Chinese SLE treatment and research group registry: III. Association of autoantibodies with clinical manifestations in Chinese patients with systemic lupus erythematosus. J. Immunol. Res..

[43] Gómez-Puerta JA, Burlingame RW, Cervera R (2008). Anti-chromatin (anti-nucleosome) antibodies: Diagnostic and clinical value. Autoimmun. Rev..

[44] Muller S, Dieker J, Tincani A, Meroni PL (2008). Pathogenic anti-nucleosome antibodies. Lupus.

[45] Migliorini P, Ardman B, Kaburaki J, Schwartz RS (1987). Parallel sets of autoantibodies in MRL-lpr/lpr mice. An anti-DNA, anti-SmRNP, anti-gp70 network. J. Exp. Med..

[46] Migliorini P, Baldini C, Rocchi V, Bombardieri S (2005). Anti-Sm and anti-RNP antibodies. Autoimmunity.

[47] Hoffman IE, Peene I, Meheus L, Huizinga TW, Cebecauer L, Isenberg D, De Bosschere K, Hulstaert F, Veys EM, De Keyser F (2004). Specific antinuclear antibodies are associated with clinical features in systemic lupus erythematosus. Ann. Rheum. Dis..

[48] Choi MY, Clarke AE, Urowitz M, Hanly J, St-Pierre Y, Gordon C, Bae SC, Romero-Diaz J, Sanchez-Guerrero J, Bernatsky S, Wallace DJ, Isenberg D, Rahman A, Merrill JT, Fortin PR, Gladman DD, Bruce IN, Petri M, Ginzler EM, Dooley MA, Fritzler MJ (2022). Longitudinal analysis of ANA in the systemic lupus international collaborating clinics (SLICC) inception cohort. Ann. Rheum. Dis..

